# Secondary Hemophagocytic Lymphohistiocytosis in Post-COVID-19 Patients: A Report of Two Cases

**DOI:** 10.7759/cureus.17328

**Published:** 2021-08-20

**Authors:** Pranjal Kalita, Devina Laishram, Biswajit Dey, Jaya Mishra, Bhupen Barman, Himesh Barman

**Affiliations:** 1 Pathology, North Eastern Indira Gandhi Regional Institute of Health and Medical Sciences, Shillong, IND; 2 Internal Medicine, North Eastern Indira Gandhi Regional Institute of Health and Medical Sciences, Shillong, IND; 3 Paediatrics, North Eastern Indira Gandhi Regional Institute of Health and Medical Sciences, Shillong, IND

**Keywords:** covid-19, secondary hemophagocytic lymphohistiocytosis, immune system, sars-cov-2, bone marrow

## Abstract

Hemophagocytic lymphohistiocytosis (HLH) is a disease that can affect both children and adults. HLH can be categorized as primary or secondary. Secondary HLH (sHLH) may be secondary to various viral infections. Severe acute respiratory syndrome coronavirus 2 (SARS-CoV-2) virus infection is a pandemic with multi-system involvement. HLH in COVID-19 positive patients is a recognized entity. However, in post-COVID-19 patients who have recovered and are negative by serological tests and reverse transcription-polymerase chain reaction test may present with sHLH due to dysregulation of the immune system. We highlight this unusual finding of post-COVID-19 sHLH in two cases, who were diagnosed by the new revised H-score.

## Introduction

Hemophagocytic lymphohistiocytosis (HLH) is a lethal disorder of varying etiology and encompasses a wide range of diseases. Familial HLH (FHLH) and immune-related HLH constitute the primary HLH spectrum whereas infections, drug-related and transplantation, malignancies, and macrophage activation syndrome (MAS) constitute the secondary HLH (sHLH) spectrum [1]. Laboratory parameters like hypercytokinemia, marked cytopenia, hyperferritinemia, hypertriglyceridemia, and hypofibrinogenemia along with clinical findings of organomegaly, lymphadenopathy, multiorgan dysfunction, and fever predominate in HLH patients [2]. Various viral, bacterial, fungal, and protozoan organisms are implicated in the etiopathogenesis of sHLH [1,2]. Severe acute respiratory syndrome coronavirus-2 (SARS-CoV-2) virus has been implicated as a causative agent of sHLH in COVID-19 positive patients; however, cases of sHLH in recovered post-COVID patients are rare. We report two cases of post-COVID-19 patients presenting with sHLH.

## Case presentation

Case 1

**Figure 1:**
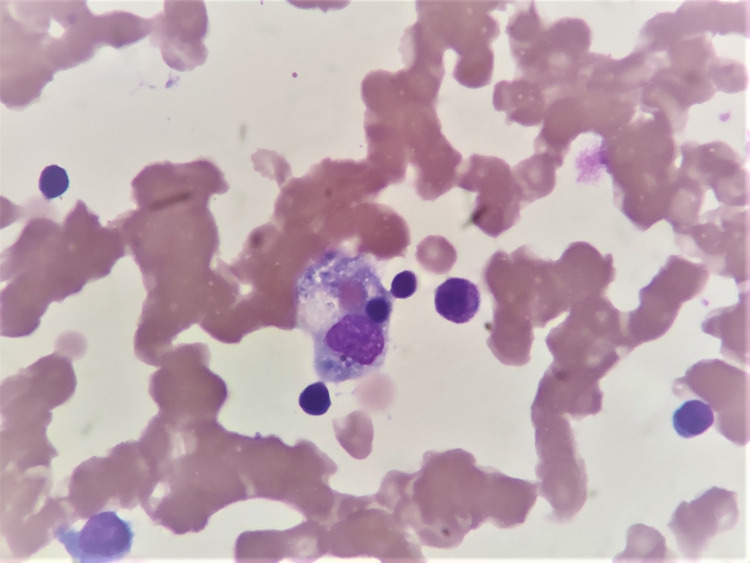
Bone marrow aspirate showing hemophagocytosis (Leishman, 1,000x)

A 40-year-old female presented with complaints of on and off episodes of fever and cough for one week and multiple episodes of the passage of loose stool for three days prior to admission. There was no history of any known co-morbidities. The patient was tested positive for COVID-19 by reverse transcription-polymerase chain reaction (RT-PCR) and was hospitalized for 1.5 months. At presentation, the patient was conscious, alert, and oriented. Her blood pressure was 96/58 mmHg, pulse rate was 118 beats per minute, respiratory rate was 33/min and SPO_2_ was 88%, and the temperature was 40̊C. On examination, pallor was present. Bilateral crepitations and rhonchi were present in respiratory system auscultation. The cardiovascular system was unremarkable and central nervous system examination showed no focal neurological deficit. Ultrasonography of the whole abdomen revealed hepatosplenomegaly. High-resolution computed tomography (HRCT) thorax showed CO-RADS category 6 and CT severity of 15/25. Bone marrow aspirate showed evidence of hemophagocytosis (Figure 1).

Laboratory investigations revealed hemoglobin (Hb) of 3.3 g%, total leukocyte count (TLC) of 18.3x10^3^ /µL with differential leukocyte count (DLC) of N87L10M03E0, and platelet count of 20x10^3^ /µL. Biochemical investigations revealed serum urea of 61 mg/dL (reference range [RR]: 17-45 mg/dL), serum creatinine of 0.9 mg/dL (RR: 0.6-1.4 mg/dL), D-dimer of 1.58 mg/L (RR: 0-0.5 mg/L), aspartate aminotransferase (AST) of 76 IU/L (RR: 5-40 IU/L), alanine aminotransferase (ALT) of 98 IU/L (RR: 5-55 IU/L), alkaline phosphatase (ALP) of 137 IU/L (RR: 20-140 IU/L), total bilirubin of 2.5 mg/dL (RR: up to 1.2 mg/dL), direct bilirubin of 1.3 mg/dL (RR: 0-0.2 mg/dL), IL-6 of 11.56 pg/mL (RR: 5-15 pg/mL), fibrinogen of 2.3 g/L (RR: 2-4 g/L), vitamin B12 of 489.5 pg/mL (RR: 160-950 pg/mL), ferritin of 170.2 ng/L (RR:12-250 ng/L), and C-reactive protein of 77mg/L (RR: 0-10 mg/L). Direct Coombs test was negative and indirect cold antibodies titer was more than 256 (RR: less than 164). All viral markers, anti-nuclear antibody (ANA), and glucose-6-phosphate dehydrogenase (G6PD) were negative. The H-score estimated as per the H-score of 2014 was 213 points with a probability of sHLH to be 93% to 96% (Table 1).

**Table 1:** Comparison of H-score-2014 in case 1 and case 2 sHLH: Secondary hemophagocytic lymphohistiocytosis

Parameters	Number of points (criteria for scoring)	Case 1	Case 2
.Known underlying immunosuppression	0 (no) or 18 (yes)	18	18
Temperature (°C)	0 (<38.4), 33 (38.4–39.4), or 49 (>39.4)	49	49
Organomegaly	0 (no), 23 (hepatomegaly or splenomegaly), or 38 (hepatomegaly and splenomegaly)	38	00
No. of cytopenias	0 (1 lineage), 24 (2 lineages), or 34 (3 lineages)	24	24
Ferritin (ng/mL)	0 (<2,000), 35 (2,000–6,000), or 50 (>6,000)	00	00
Triglyceride (mmoles/liter)	0 (<1.5), 44 (1.5–4), or 64 (>4)	00	64
Fibrinogen (g/L)	0 (>2.5) or 30 (≤2.5)	30	30
Serum glutamic oxaloacetic transaminase (IU/L)	0 (<30) or 19 (≥30)	19	19
Hemophagocytosis features on bone marrow aspirate	0 (no) or 35 (yes)	35	35
Total H-score		213	239
Probability of sHLH		93% to 96%	98% to 99%

According to the protocols of our institute, the patient was isolated and started on intravenous steroids (dexamethasone acetate 10 mg/day), low molecular weight heparin (LMWH), antibiotics, and oxygen support. Considering the deranged kidney function, strict monitoring and adequate hydration were provided along with four units of whole blood transfusion. The patient developed congestive cardiac failure during the hospital stay and oral diuretics were initiated. The patient responded to treatment without the addition of etoposide and was hemodynamically stable. She was discharged after four weeks of hospital stay. The patient was reviewed after 14 days and was stable.

Case 2

**Figure 2:**
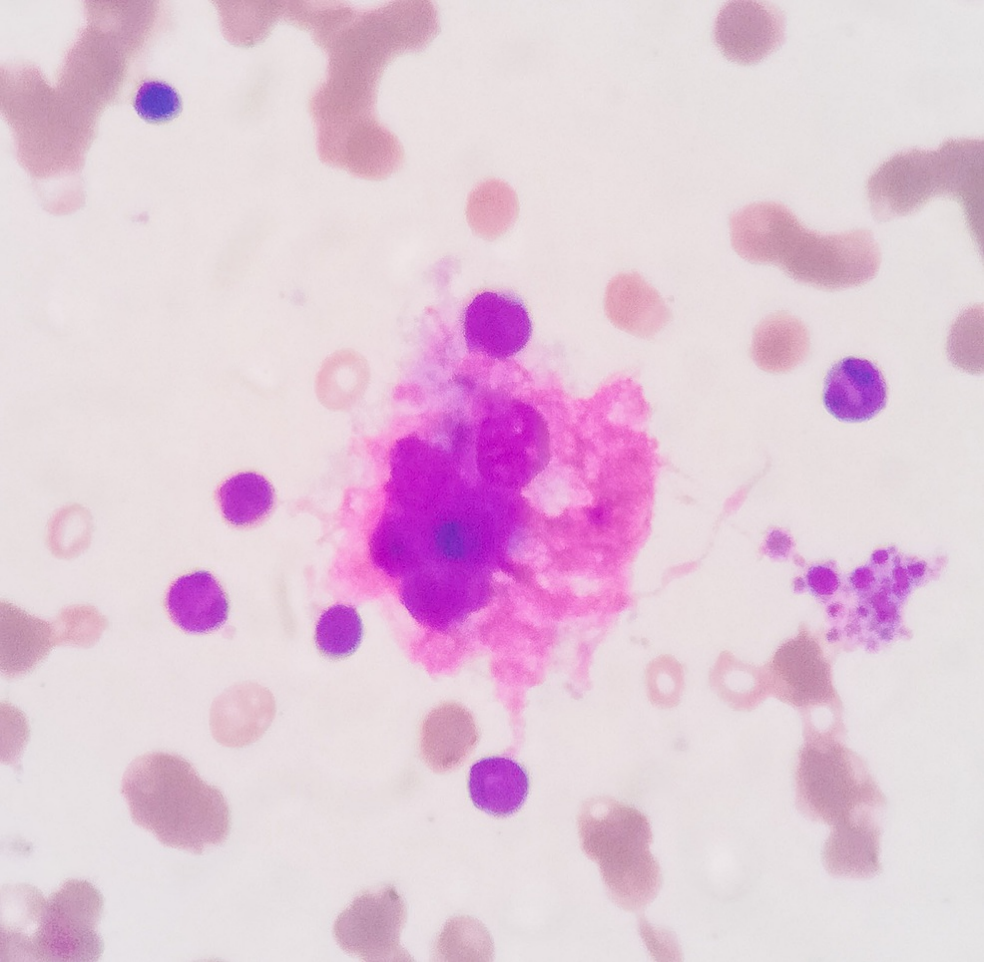
Bone marrow aspirate showing hemophagocytosis (Leishman, 1,000x)

A 2-year-old male weighing 9 kg presented with complaints of abnormal body movements along with feeding intolerance, fever (39.6̊C), diarrhea, and vomiting for two days. The patient had no previous episodes in the past and developmental milestones were achieved at his age normally. On examination of muscle tone, neck control was decreased. A possible diagnosis of post-viral encephalitis was considered. The patient was positive for SARS-CoV-2 virus by RT-PCR and was admitted to COVID-19 designated hospital. He was discharged after testing negative for the SARS-CoV-2 virus. After two weeks of discharge, he presented with the above symptoms but was negative for COVID-19 by RAT and RT-PCR. MRI brain showed features suggestive of viral encephalitis with atrophy of cerebral hemispheres and peritrigonal white matter hyperintensities. Laboratory findings revealed Hb of 7.1 g%, TLC of 7.3x10^3^ /µL with DLC N86L10M02E02, and platelet count of 60x10^3^ /µL. Biochemical investigations showed serum ferritin of 188.0 ng/mL (RR: 7-140 ng/mL), IL-6 of 1,057 pg/mL (RR: 1-5 pg/mL), D-dimer of 1.76 mg/L (RR: 0-0.5 mg/L), AST of 182 IU/L (RR:10-40 IU/L), ALT of 77 IU/L (RR:10-40 IU/L), ALP of 77 IU/L (RR: <350 IU/L), C-reactive protein of 62.1 mg/L (RR: 0.02-14.5 mg/L), and fibrinogen of 2.13 g/L (RR: 2-4 g/L). Malaria, scrub typhus, HBsAg, anti-HCV were negative. Bone marrow aspirate revealed features of hemophagocytosis with increased iron (Figure 2).

The H-score estimated as per the H-score of 2014 was 239 points with a probability of sHLH to be 98% to 99% (Table 1). The patient was treated in the pediatric intensive care unit (PICU) and anti-epileptics, antibiotics, fluids, and steroids were started. The patient was being monitored in PICU at the time of submission of the manuscript.

## Discussion

HLH was first described by Farquhar and Claireaux in the year 1952 [3]. HLH is characterized by a “cytokine storm” caused by uncontrolled activation of cytotoxic T-lymphocytes, and natural killer (NK) cells leading to increased secretion of IFN-γ, TNF-α, and IL-1ß,2,6,12,16 which ultimately turns macrophage hyperactive [4]. Primary HLH is primarily a genetic disorder manifesting mainly in children whereas sHLH is a disease primarily of the adult with multiple etiologies [5]. sHLH may occasionally be a complication of various infections and viral infections are one of the common culprits for sHLH [6]. The recent pandemic of the SARS-CoV-2 virus has highlighted that sHLH in COVID-19 positive patients may result in a considerable increase in mortality [6]. Although cases of sHLH in active COVID-19 positive patients are increasingly recognized; however, sHLH in post-COVID-19 recovered patients is sparse in the literature [5]. A prompt diagnosis of HLH is of utmost importance as drugs like etoposide administered at the early stage may improve survival [7].

HLH is a relatively underdiagnosed entity with an absence of definitive clinical, laboratory, or histopathological criteria [8]. Hemophagocytic activity in the bone marrow aspirate is considered to be a sign of HLH; however, such hemophagocytic activities are also noted in critically septic patients or patients who have received a transfusion [8-10].

The HLH-1994 criteria included various clinical (fever, splenomegaly), laboratory (cytopenias, hypertriglyceridemia, and/or hypofibrinogenemia), and histological parameters (hemophagocytosis in bone marrow or spleen or lymph nodes with no evidence of malignancy), which was modified in 2004 [11]. The HLH-2004 included additional parameters like low or absent NK cell activity, serum ferritin, and IL-2 receptor level for aiding in the diagnosis. The HLH-2004 study group notes that all diagnostic criteria are sometimes not met or few of the diagnostic criteria develop late in the course of the disease, hence it’s recommended to consider a diagnosis of HLH and start treatment based on clinical suspicion. The H-score devised by Fardet et al. was a modification of the previous systems and gave simplified numerical values to the various clinical, laboratory, and histological parameters which is comparatively easier and helps in prompt diagnosis of sHLH patients [12].

The optimal cut-off for a diagnosis of HLH was considered to be 169 by Fardet et al. with 93% sensitivity and 86% specificity [13]. In our first case, the H-score was 213 points which predict a 93% to 96% probability of hemophagocytic syndrome whereas in the second case the H-score was 239 points which predict a 98% to 99% probability of hemophagocytic syndrome. Thus, a diagnosis of sHLH was considered in both the patients in view of the high revised H-score, clinical, laboratory, and histological findings.

HLH in COVID-19 positive patients is reported in various case reports [14,15]. However, the cases of sHLH in patients who have recovered from COVID-19 and are RAT and RT-PCR negative are sparse. Naous et al. reported a case of HLH as a cause of death in the post-COVID-19 patient [5]. Persistent subclinical inflammation and a latent state may be implicated as causation of such presentation [5]. The two cases described presented in our institute with symptoms that lead to clinical suspicion of sHLH and H-score further confirmed the diagnosis of sHLH in our patients. Dysregulation of the immune system secondary to COVID-19 infection may be a possible cause of sHLH in post-COVID-19 patients. A differential diagnosis of sHLH is to be always considered in such post-COVID-19 patients in spite of limited literature supporting the same.

## Conclusions

SARS-CoV-2 infection is a recent pandemic with important complications inherent to its multisystemic affections. Recent information about etiopathogenesis, clinical signs and symptoms, and treatment protocols are getting highlighted. sHLH is a condition primarily occurring in COVID-19 positive patients; however, sHLH in post-COVID-19 patients is rare. Immune dysregulation following infection by the SARS-CoV-2 virus may be the prime reason for sHLH in such patients. We highlighted two such post-COVID-19 patients who were negative for COVID-19 at the time of presentation but were diagnosed as cases of sHLH based on the H-score.
